# A decade of urban fires: Portuguese events between 2013 and 2022

**DOI:** 10.1038/s41597-023-02476-6

**Published:** 2023-08-26

**Authors:** Regina Bispo, Filipe J. Marques, Alexandre Penha, Pedro Espadinha-Cruz, António Grilo

**Affiliations:** 1 Center for Mathematics and Applications (NOVAMath) and Department of Mathematics, NOVA School of Science and Technology, Universidade NOVA de Lisboa, 2829-516 Caparica, Portugal; 2 Comando Nacional de Emergência e Proteção Civil, ANEPC Autoridade Nacional de Emergência e Proteção Civil, 2794-112 Carnaxide, Portugal; 3 UNIDEMI, Department of Mechanical and Industrial Engineering, NOVA School of Science and Technology, Universidade NOVA de Lisboa, 2829-516 Caparica, Portugal

**Keywords:** Scientific data, Society

## Abstract

This study describes a dataset containing urban fire events that took place in mainland Portugal between 2013 and 2022. The Regulation n.º3317-A/2018, established by the Portuguese National Emergency and Civil Protection Authority (*Autoridade Nacional de Emergência e Proteção Civil*, ANEPC), defines the *Operations Management System* (*Sistema de Gestão de Operações*, SGO). Among other attributions, this system allows to manage the lyfe-cycle of the urban fire events, from ignition to extinction, through the *Operations Decision Support System* (*Sistema de Apoio Ã Decisão Operacional*, SADO). This system supports the systematic collection of a minimum set of data on each event. All instances included in the dataset were retrieved from SADO. To make the data suitable for analytic purposes, several pre-processing actions were taken, including the steps of data transformation and cleaning. The dataset was further validated by a set of technical procedures aiming to verify both data correctness and utility. The final dataset provides the most recent multi-year record of Portuguese urban fires including 27 variables on 72641 events.

## Background & Summary

Urban Firefighting is a major theme on the scientific field of Emergency Management, generically aiming at (1) finding ways to act in face of urban fires and (2) address mitigation measures for safer communities. In Portugal, Emergency Management and Fire Fighting is governed by the Portuguese National Emergency and Civil Protection Authority (*Autoridade Nacional de Emergência e Proteção Civil*, ANEPC). Provided emergency services are mostly funded by public budgets. This coupled with the autonomy of local firefighter associations for decision making leads to a rather complex and fragmented management of the existing urban firefighting resources, potentially involving several entities such as, e.g., local firefighter associations, police, medical services, insurances^1^. These constraints have resulted in an heterogeneous and highly asymmetrical urban firefighting response. Over the years, Portugal has experienced sporadic but significant urban fire incidents, characterized by their severity and impact. For example, in 1988, the Chiado neighborhood in the center of Lisboa, Portugal, was severely affected by a fire. Firefighting included a total of 1680 firefighters and affected a total of 18 buildings of which 11 were complete losses. Two people were killed and 73 were injured. Hundreds of people lost their homes, and thousands lost their jobs^2^. Another Portuguese urban fire with severe consequences happened more recently, in 2018, in Tondela, and also resulted in severe human injures and loss of lives. These events clearly illustrate the dire consequences that can arise from urban fires including human casualties and injuries, direct losses of houses, structures, equipments, and other properties^3^ and indirect consequences such as people displacement and employment loss. They also serve as powerful reminders of the ongoing need for improved fire prevention and building safety standards, particularly in high-risk areas. Furthermore, because in cities the buildings and population densities are high, there is a growing need in effective fire risk management^4^. Within fire risk management, fire risk analysis plays a fundamental role^5^ as it provides information regarding high-risk areas and may guide fire preventive measures.

To assess and manage fire risks, appropriate modelling approaches of fire events are necessary. Recent literature in the field of urban firefighting has revisited various conceptual theories to model fires occurrence based on, e.g., building features and/or social-economic characteristics^6–8^.Kumar *et al*.^9^ claimed that different methodologies may provide additional insights into fire management by revealing trends, patterns, and hidden information that otherwise would pass unnoticed. Recently, Jin *et al*.^8^ underlined that the trend in urban fires modelling has shifted from mathematical based to data-driven statistical learning approaches. In particular, Turner *et al*.^10^ examined the use of machine learning techniques to identify risk factors for unintentional house fires. Other current research trend focuses on the spatio–temporal analysis which provides a framework for understanding the distribution, patterns, and dynamics of fires^11^. Despite these methodological advances, there is still a dearth of available information in particular with regard to urban fires^12,13^, which often leads to unreliable results, making it more challenging to adopt and apply evaluation tools in different urban contexts. However, obtaining comprehensive and organized fire data is frequently complicated, since generally requires the participation of local authorities and emergency management and firefighting institutions^14^. Further, raw information is often plagued by poor quality^15^. Dey *et al*.^15^ cite various examples, such as, the utilization of inconsistent abbreviations and acronyms to identify properties, discrepancies in addresses, incomplete dates, and missing timestamps.

Thus, having good quality datasets regarding urban fire occurrences seems crucial in filling up this gap. This type of data represents a valuable tool as their study may provide significant insights into the spatio-temporal dynamics of urban fires, therefore serving as a foundation for to manage risks, model fire occurrence and building spatially integrated operational plans. Collecting urban fire data is a fundamental key step to characterize ignition patterns across time and space and identify factors that contribute to fire events allowing for targeted interventions and more efficient resource allocation. Moreover, accurate data may have the potential to guide future policies in the fire and rescue services. Additionally, the combination of fire data with information on, e.g., weather and social-economic factors can further enhance the understanding of urban fires dynamics. With the integration of spatial planning to manage fire risk becoming a priority for many local authorities^11^, publishing data descriptors on urban fire occurrences indexed both in time and space, such as this one, may support evidence-based policymaking playing a crucial role in promoting the safety and well-being of the communities.

Several studies were already based on at least a subset of the data described in this manuscript. In particular, Bispo *et al*.^16^ model urban fire occurrences at a municipality level, ultimately mapping the probability of urban fires occurrence in mainland Portugal, and Eslamzadeh *et al*.^17^ assessed fire departments performance using a slack-based Data Envelopment Analysis (DEA).

**Fig. 1:**
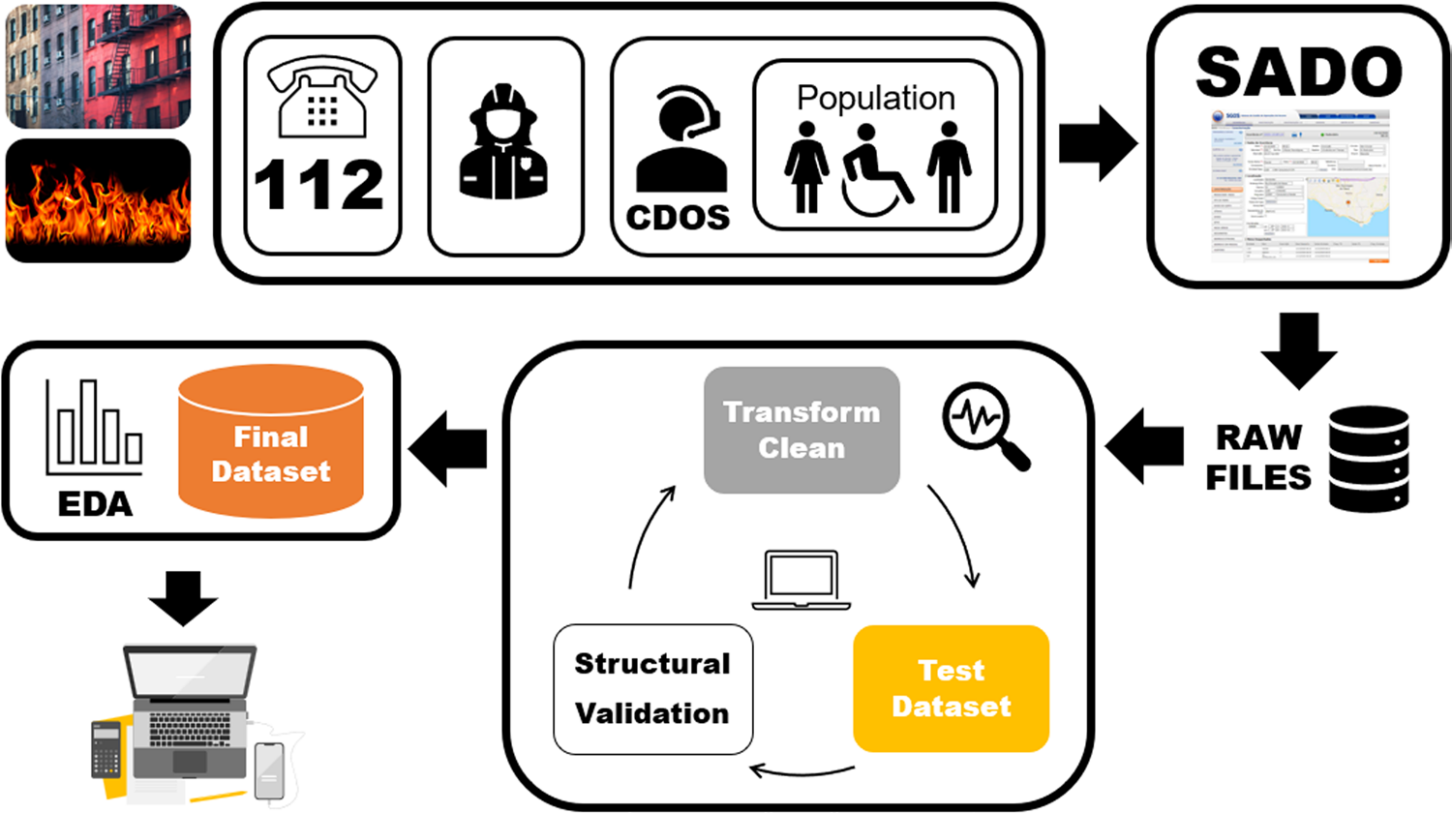
Schematic representation of the study. Information on urban fires are registered in SADO plataform directly if the alert comes from the national emergency telephone number 112 or, indirectly, through the district emergency operations command CDOS if the alert is given by the general population or from the fire departments. After registered, the occurrences were downloaded (raw data) and processed (transformed an cleaned). The final fire dataset was validated based on previous domain knowledge and exploratory data analysis (EDA) making it ready for further reuse.

This study aims at providing access to the latest Portuguese Urban Fire Database, both in their raw and clean formats, including detailed information on pre-processing actions and data validation methods. Furthermore, a comprehensive description of the dataset is provided along with the code used to analyse it. Figure 1 represents schematically the study. Raw data were retrieved from ANEPC arquive. There is no historical open access data available on ANEPC website. Only daily occurrences, incompletely reported (i.e., with no data regarding injuries or deaths), are available online. The raw data were transformed and cleaned, and then checked for correctness until pass all validation criteria, making it ready to reuse in several further contexts. In addition, as the code is made available, this study is readily updatable by any user.

The next section describes in detail the used methods. The raw and final datasets are described in the Data Records section. Finally, in the Technical validation section we address the structural and content validation procedures including an exploratory data analysis to validate data further reuse.

## Methods

The Portuguese regulation n.º3317-A/2018 establishes the National Emergency and Civil Protection System *Operations Management System* (*Sistema de Gestão de Operações*, SGO). Among other attributions, this system allows the management of emergencies, through the *Operations Decision Support System* (*Sistema de Apoio Ã Decisão Operacional*, SADO). Since 2012, this platform supports the systematic collection of a minimum set of data on each emergency event and integrates national occurrences. All events reported to ANEPC must be registered in SADO. The information on a event may arrive to ANEPC through several sources, including the direct report from the population, the national emergency telephone number 112 or the communication from the fire departments. If the alerts arrive from 112, the information is directly inserted in SADO system. For the remain type of alerts, the information is first given to the telecommunications operators of the district emergency operations command (*Comando Distrital de Operações de Socorro*, CDOS), which then are responsible to register the information in SADO. This system allows to store relevant information on the life-cycle of fire events, from ignition to extinction. Events regarding urban fires are coded by an integer between 2101 and 2130 (depending on the type of affected structure). All instances included in the dataset were extracted from SADO and exported to an .xlsx Excel file. The raw urban fire data encompassed 34 features on 72743 events that took place between 2013 and 2022.

To make the data suitable for analytic purposes, several pre-processing actions were taken, including the steps of *data transformation*, *data cleaning* and *data validation* (see details in section Technical Validation). All analyses were carried out using R^18^, an open-source software environment for statistical computing. Firstly, the .xlsx Excel file was read into an R data frame containing all retrieved objects between 2013 and 2022. Data regarding the geographical delimitation of the administrative units of Portugal were obtain from shapefiles downloaded from the open data public certified service https://dados.gov.pt/^19^ that contains the geometry of the administrative divisions of the country, namely the official boundaries of districts, municipalities, and parishes. These files are made available by the Agência para a Modernização Administrativa, I.P. (AMA), a Portuguese public institute entrusted with the task of fostering and advancing administrative modernization in Portugal, which operates under the supervision of the Secretary of State for Digitalization and Administrative Modernization. These geometry data were restricted to mainland Portugal to allow the rigorous mapping of the events. Packages raster^20^ and sf^21^ were used to read shapefiles and deal with geometry indexed objects.

The raw dataset contained two type POSIXct, 15 type character and 17 type numeric features (Table 1). The data transformation process included the following steps:

- all variables were renamed using English derived natural names, with only lowercase letters;
- the class labels of all factors (except fire departments and administrative units names) were recoded, translating the categories labels to English;
- administrative units (variables region, subregion, district, municipality and parish) were consistently written, both in fire and geometry data, using lowercase letters, followed by capitalisation;
- features with constant values were deleted from the dataset (variables state, family and species);
- the observations and locality variables were discarded as no consistent or complete information was retrievable from it;
- variables code and codefd were deleted due to bijective correspondences with type and pfd, respectively;
- variable period was deleted due to redundancy with open;
- variable pfd was recoded, replacing the string “NULL” by “Unkown”;
- type character variables were transformed to factors.

**Table 1:** Raw urban fire dataset features, including original and given names, structure and summary description.

	Raw name	New name	Type	Description
1	*Número*	id	numeric	unique reference (sequence of 13 digits) of each fire record
2	*Estado*	state	character	state of the event with one unique string
3	*Importância*	importance	character	severity score, including categories *Auto-interface*, *Elevada*, *Moderada* and *Reduzida*
4	*Início*	open	POSIXct	date (%Y-%m-%d) and time (%H:%M:%S) of first alert, i.e., at which the fire protection services were informed of the outbreak of the fire
5	*Fecho operacional*	close	POSIXct	date (%Y-%m-%d) and time (%H:%M:%S) of fire extinction, i.e., at which all fire-fighting units left the fire scene
6	*Duração (min)*	length	numeric	length of time between fire outbreak and extinction, in minutes
7	*Código*	code	character	label given by an integer between 2101 and 2130 according to the type of affected urban infrastructure (variable *Nome*)
8	*Família*	family	character	type of hazard with one unique string
9	*Espécie*	species	character	type of fire with one unique string
10	*Nome*	type	character	type of affected urban infrastructure
11	*Região*	region	character	Portuguese region where the fire started (NUTS II regions administered by the Commissions for Coordination and Regional Development), with 5 unique strings
12	*SubRegião*	subregion	character	Portuguese subregion where the fire started administrative subregions (NUTS III regions administered by the Commissions for Coordination and Regional Development), with 23 unique strings
13	*Distrito*	district	character	Portuguese district where the fire started
14	*Concelho*	municipality	character	Portuguese municipality where the fire started
15	*Freguesia*	parish	character	Portuguese parish where the fire started
16	*Localidade*	locality	character	address details
17	*Latitude*	lat	numeric	latitude of the fire ignition location
18	*Longitude*	lon	numeric	longitude of the fire ignition location
19	*Código Operacional*	codefd	numeric	label given by an integer representing the primarily responsible fire department
20	*Entidade Responsável*	pfd	character	primarily fire department responsible for the event
21	*Humanos Terr*	groundhr	numeric	number of firefighters (ground firefighting)
23	*Humanos Aer*	airhr	numeric	number of firefighters (air firefighting)
22	*Técnicos Terr*	groundtr	numeric	number of technical resources (ground firefighting)
24	*Técnicos Aer*	airtr	numeric	number of technical resources (air firefighting)
25	*Mortos*	deaths	numeric	number of human fatalities
26	*Feridos Graves*	major	numeric	number of major human injuries
27	*Feridos Ligeiros*	minor	numeric	number of minor human injuries
28	*Assistidos*	assist	numeric	number of human assistances
29	*Feridos Outros*	other	numeric	number of other human victims (including missing, homeless, displaced, evacuated and buried)
30	*Vítimas APC*	apc	numeric	total number of non-civilian victims
31	*Outras Vítimas*	otherv	numeric	total number of civilian victims
32	*Reacendimento*	reign	character	reignition of fire that previously has been given as extinguished, with two unique unique strings
33	*Periodo*	period	character	period of the day in which the fire took place with two unique unique strings
34	*Descrição*	observations	character	addition details (if available)

At this stage, the dataset included 72743 objects and 26 features (initial 34 minus 8). After transformation, the data were screened for a set of structural validation rules (see section Technical validation). The dataset was then cleaned to correct the detected errors and, subsequently, submitted to a content validation (see section Technical validation) that allowed to improve/update the data transformation and cleaning steps in an iterative way.

## Data Records

The urban fire data between 2013 and 2022 were retrieved from SADO. The raw data organized in an.xlsx Excel file (rawdata.xlsx), containing high-level features for each fire record, are available at figshare^22^. Table 1 summarizes the raw dataset features including the original (raw) and given (new) names, the respective type and a summary description for each feature. Table 2 details the final dataset after processing and validating the initial data, as described in the Methods and Tecnhical Validation sections. The final dataset which provides the most recent multi-year record of Portuguese urban fires including 27 variables on 72641 events (finaldata.xlsx) is available at figshare^22^.

**Table 2:** Final urban fire dataset features, including original and given names, structure and summary description.

	Raw name	New name	Type	Description
1	*Número*	id	numeric	unique reference (sequence of 13 digits) of each fire record
2	*Importância*	importance	factor	severity score, including categories *unknown*, if no information is given, *high* if length is greater or equal to 60 minutes or there are at least 10 victims or at least 1 death, *low* length is under 30 minutes and there are less than 10 victims and 0 deaths, and *medium* otherwise.
3	*Início*	open	POSIXct	date (%Y-%m-%d) and time (%H:%M:%S) of first alert, i.e., at which the fire protection services were informed of the outbreak of the fire
4	*Fecho operacional*	close	POSIXct	date (%Y-%m-%d) and time (%H:%M:%S) of fire extinction, i.e., at which all fire-fighting units left the fire scene
5	*Duração (min)*	length	numeric	length of time between fire outbreak and extinction, in minutes
6	*Nome*	type	factor	type of affected urban structure, including categories *Residential*, *Above surface parking*, *Underground parking*, *Administrative services*, *School campus*, *Hospitals and Nursing homes*, *Shows and Public events*, *Hotels and Restaurants*, *Commercial areas and Transport stations*, *Sports and Leisure centers*, *Museums and Art galleries*, *Libraries and Archives*, *Military, security and emergency forces*, *Industry, Workshops and Warehouses* and *Degraded or unoccupied buildings*
7	*Região*	region	factor	Portuguese region where the fire started (NUTS II regions administered by the Commissions for Coordination and Regional Development), with 5 unique strings
8	*SubRegião*	subregion	factor	Portuguese subregion where the fire started administrative subregions (NUTS III regions administered by the Commissions for Coordination and Regional Development), with 23 unique strings
9	*Distrito*	district	factor	Portuguese district where the fire started
10	*Concelho*	municipality	factor	Portuguese municipality where the fire started
11	*Freguesia*	parish	factor	Portuguese parish where the fire started
12	*Latitude*	lat	numeric	latitude of the fire ignition location
13	*Longitude*	lon	numeric	longitude of the fire ignition location
14	*Entidade Responsável*	pfd	factor	primarily fire department responsible for the event
15	*Humanos Terr*	groundhr	numeric	number of firefighters (ground firefighting)
16	*Humanos Aer*	airhr	numeric	number of firefighters (air firefighting)
17	*Técnicos Terr*	groundtr	numeric	number of technical resources (ground firefighting)
18	*Técnicos Aer*	airtr	numeric	number of technical resources (air firefighting)
19	*Mortos*	deaths	numeric	number of human fatalities
20	*Feridos Graves*	major	numeric	number of major human injuries
21	*Feridos Ligeiros*	minor	numeric	number of minor human injuries
22	*Assistidos*	assist	numeric	number of human assistances
23	*Feridos Outros*	other	numeric	number of other human victims (including missing, homeless, displaced, evacuated and buried)
24	*Vítimas APC*	apc	numeric	total number of non-civilian victims
25	*Outras Vítimas*	otherv	numeric	total number of civilian victims
26	*Reacendimento*	reignit	factor	reignition of fire that previously has been given as extinguished, including categories *no* and *yes*
27		cty	factor	type of spatial coordinates, including categories *not exact* if the fire ignition point is given by the parish centroid and *exact*, otherwise

## Technical Validation

The purpose of this data descriptor is to offer a wealth of information that can be used to gain new insights, perform detailed analyses, and uncover patterns and trends in urban fire dynamics that, otherwise, would not be possible to study. To achive this goal, a dataset technical validation is mandatory.

Data validation may be generically defined as the process of analysing the quality of data and deciding whether if it satisfies the assumptions based on domain knowledge and if fits the purpose^23^. Furthermore, according to the *European Statistical System* data validation is the “*activity aimed at verifying whether the value of a data item comes from the given (finite or infinite) set of acceptable values*”^24^. In this study, the data were checked iteratively for correctness until the final dataset met the desired quality. This data validation process included two steps, in which the second allowed to review the output from the first, improving the validation procedure in an iterative way. The two steps defined, by order, a (1) *structural validation* in which data were checked against a set of criteria and the validation results were measured, using R package validate^23^, and a (2) *content validation* in which the dataset was statistically reviewed to improve/update the list of validation rules and ultimately guaranty the desired dataset quality standards. The former was carried out by checking the correctness of each feature based on univariate and multivariate validation rules. The latter was based on descriptive statistics and exploratory data analysis.

### Structural validation

Table 3 summarizes the validation rules. Two types of validation rules were employed: (1) univariate, defined according to the set of acceptable domains for each feature and applied independently to each one of the variables included in the dataset, and (2) multivariate, involving relationships between variables. Univariate validation rules included checks regarding class attribute for all variables. In addition, for numerical and time/date features, we included checks about the values range, and for factors, checks based on code lists containing allowed values. Multivariate validation rules were applied to variables importance, close, length, region, subregion, district, municipality, parish, deaths, major, minor, assist, other, apc and otherv. A total of 67 initial structural validation rules were defined from which the following 13 failed (rules identification according to Table 3):

- *low* importance if [deaths = 0] ∧ [(major + minor) < 5] ∧ [length < 30] (rule [${r}_{5}$], 26 cases out of 72743);
- *high* importance if [deaths ≥1] ∨ [(major + minor) ≥10] ∨ [length ≥60] (rule [${r}_{6}$], 35106 cases out of 72743);
- *medium* importance (rule [${r}_{7}$], 29159 cases out of 72743);
- close > open (rule [${r}_{12}$], 6 cases out of 72743)
- positive length (rule [${r}_{14}$], 6 cases out of 72743);
- length equal to difference between close and open (rule [${r}_{15}$], 795 cases out of 72743);
- match district retrieved from (lon, lat); (rule [${r}_{28}$], 120 cases out of 72743)
- municipality levels in code list given by geometry data (rule [${r}_{31}$], 114 cases out of 72743);
- match municipality retrieved from (lon, lat) (rule [${r}_{32}$], 448 cases out of 72743);
- parish levels in code list given by geometry data (rule [${r}_{35}$], 33340 cases out of 72743);
- parish retrieved from (lon, lat) (rule [${r}_{36}$], 34550 cases out of 72743);
- latitude (simple decimal standard coordinates) ≥36.96 and ≤42.15 (rule [${r}_{38}$], 6 cases out of 72743);
- longitude (simple decimal standard coordinates) ≥−9.55 and ≤−6.19 (rule [${r}_{40}$], 3 cases out of 72743).

**Table 3:** Dataset validation rules.

Feature	Rules
id	[${r}_{1}$] class attribute = numeric, [${r}_{2}$] uniqueness
importance	[${r}_{3}$] class attribute = factor, [${r}_{4}$] levels in code list
	[${r}_{5}$] equal to *low* if [deaths = 0] ∧ [(major + minor) < 5] ∧ [length < 30]
	[${r}_{6}$] equal to *high* if [deaths ≥1]∨ [(major + minor) ≥10] ∨ [length ≥ 60]
	[${r}_{7}$] equal to *medium* otherwise
open	[${r}_{8}$] class attribute = POSIXct, [${r}_{9}$] from “2012-01-01 00:00:00” to “2022-12-31 23:59:00”
close	[${r}_{10}$] class attribute = POSIXct, [${r}_{11}$] from “2012-01-01 00:00:00” to “2023-01-01 23:59:00”
	[${r}_{12}$] close > open
length	[${r}_{13}$] class attribute = numeric, [${r}_{14}$] positive
	[${r}_{15}$] equal to difference between close and open
type	[${r}_{16}$] class attribute = factor, [${r}_{17}$] number of levels $\le$ number of levels in code list, [${r}_{18}$] levels in code list
region	[${r}_{19}$] class attribute = factor, [${r}_{20}$] number of levels ≤ number of levels given by geometry data, [${r}_{21}$] levels in code list given by geometry data
subregion	[${r}_{22}$] class attribute = factor, [${r}_{23}$] number of levels ≤ number of levels given by geometry data, [${r}_{24}$] levels in code list given by geometry data
district	[${r}_{25}$] class attribute = factor, [${r}_{26}$] number of levels ≤ number of levels given by geometry data, [${r}_{27}$] levels in code list given by geometry data
	[${r}_{28}$] match district retrieved from (lon, lat)
municipality	[${r}_{29}$] class attribute = factor, [${r}_{30}$] number of levels ≤ number of levels given by geometry data, [${r}_{31}$] levels in code list given by geometry data
	[${r}_{32}$] match municipality retrieved from (lon, lat)
parish	[${r}_{33}$] class attribute = factor, [${r}_{34}$] number of levels ≤ number of levels given by geometry data, [${r}_{35}$] levels in code list given by geometry data
	[${r}_{36}$] match parish retrieved from (lon, lat)
lat	[${r}_{37}$] class attribute = numeric, [${r}_{38}$] ≥ 36.96 and ≤ 42.15??
lon	[${r}_{39}$] class attribute = numeric, [${r}_{40}$] ≥−9.55 and ≤−6.19??
pfd	[${r}_{41}$] class attribute = factor
groundhr, airhr, groundtr, airtr, deaths, minor, major, assist, other, apc, otherv	[${r}_{42}$ to ${r}_{52}$] class attribute = numeric, [${r}_{53}$ to ${r}_{63}$] positive
	[${r}_{64}$] (deaths + major + minor + assist + other) = (apc + otherv)
reignit	[${r}_{65}$] class attribute = factor, [${r}_{66}$] number of levels ≤ number of levels given by code list, [${r}_{67}$] levels in code list
cty	[${r}_{68}$] class attribute = factor, [${r}_{69}$] levels in code list

As a consequence, the following cleaning actions were taken:

1. variable length was replaced by the time difference between the timestamps of fire extinction and fire outbreak (close and open variables);
2. cases with negative fire lengths were removed from the dataset;
3. variable importance was retrieved from length, deaths, major and minor according to the respective definition (see Table 2);
4. spatial coordinates (lat and lon) were restricted to Portuguese borders;
5. the number of levels and the names of the administrative units were retrieved from geometry data;
6. district labels were retrived from geometry data according to fire ignition coordinates (lat and lon variables);
7. municipality labels were retrived from geometry data according to fire ignition coordinates (lat and lon variables);
8. parish labels were retrived from geometry data according to fire ignition coordinates (lat and lon variables).

**Fig. 2:**
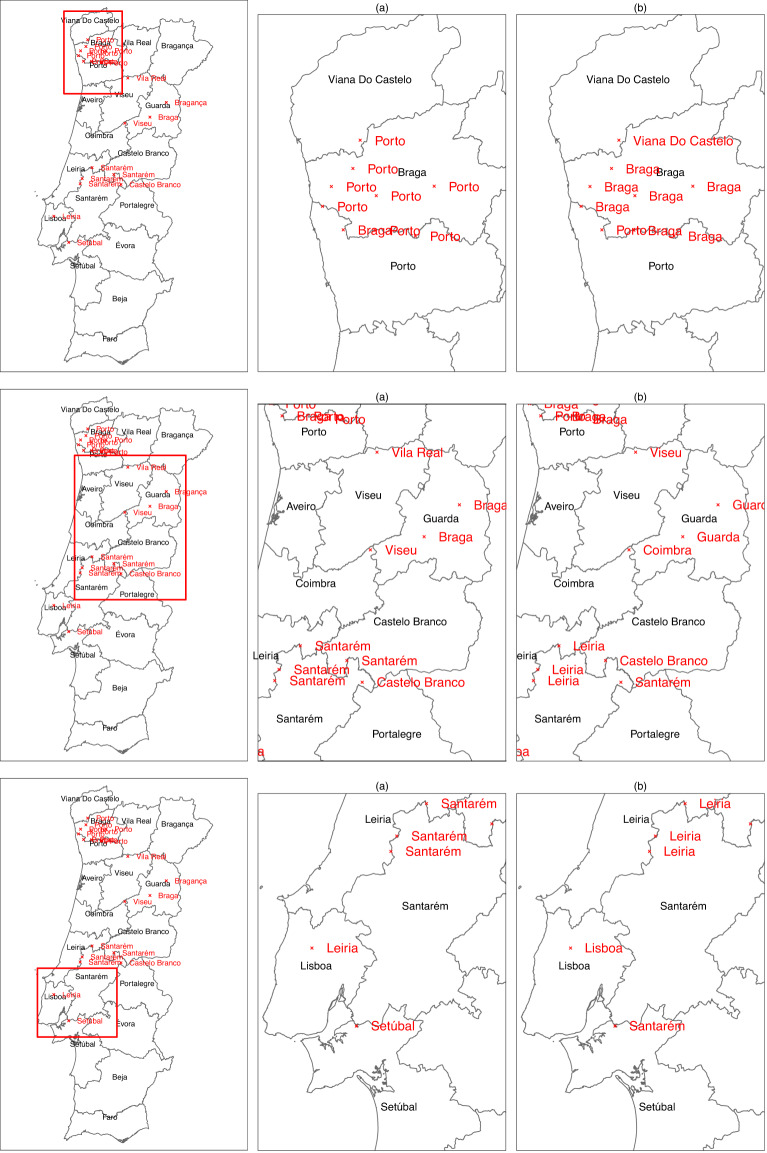
District labels correction: (**a**) Before correction and (**b**) After correction (true labels in red).

**Fig. 3:**
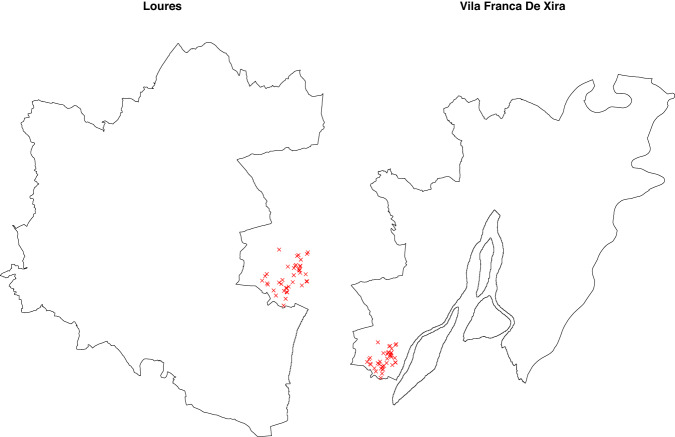
Example of municipality mislabelled objects (red points). Events that were given as belonging to *Loures* parish and fall outside this municipality (left map) belong to *Vila Franca de Xira* municipality (right map).

At this stage the dataset included 72743 cases and 26 variables. Action **(2)** excluded 7 (0.01%) cases (6 cases with negative length plus one missing value). Action **(3)** corrected 26 (0.04%) *low importance*, 29159 (40.1%) *medium importance* and 35106 (48.3%) *high importance* entries. Action **(4)** excluded 95 (0.13%) objects (outside Portuguese borders). Action **(5)** allowed to correct one municipality name (missing accent mark). Finally, actions **(6)** to **(8)**, corrected 25 (0.034%) mislabelled objects regarding their district, 239 (0.329%) mislabelled events regarding municipality and 1107 (1.522%) mislabelled objects regarding parish. These errors were confirmed by mapping the coordinates of mislabelled administrative units and checking visually that points fell outside the given administrative unit. Correction was carried out by spatial overlay between the dataset coordinates and the geometry data, finding for each data entry the true geometric unit in which each spatial point fell. This was done for all districts, municipalities and parishes, followed by mapping coordinates to validate the correction and confirm visually that points were labelled under the correct administrative unit names. Figure 2 shows the corrections regarding the district labels, whereas Fig. 3 exemplifies the municipality name correction between *Loures* and *Vila Franca de Xira* municipalities.

At this stage the dataset included 72641 cases and 26 variables. No missing values were detected, all the validation rules passed and we proceeded with the content validation, which is detailed in the following subsection.

### Content validation

In this section we present the content validation of the dataset by means of descriptive data analysis aiming to (1) update (if needed) the validation rules used in the structural validation, and (2) ultimately, by properly describing the data, guaranty the desired dataset quality standards.

### Initial summary statistics

The first step towards content validation included verifying the summary statistics for all the variables to check if the initial structural validation was enough to guaranty the desired data quality. This analysis showed suspicious ranges for variables length, other (number of other human injuries) and otherv (total number of civilian victims) with maximum values of 2.9 years, 900 and 915, respectively.

All data entries with length above 1 day were checked individual and manually. A set of 173 entries were found to be incorrect regarding variable length. As a consequence, these values and the corresponding closing dates/times were replaced by NA values (approximately 0.24%).

Entries regarding the total number of civilian victims were checked and found to be correct. These figures reflect the evacuation of key infrastructure sites or those in close proximity to them. Examples of such critical infrastructure include schools, places of worship, and commercial areas. The majority of these numbers are directly linked to the displacement of individuals, i.e., those who have been evacuated from these sites.

In addition, 363 entries were found to have simultaneously zero firefighters and technical resources (variables groundhr, airhr, groundtr and airtr), which is not possible. These were all replaced by NA values.

### Exploratory data analysis

**Fig. 4:**
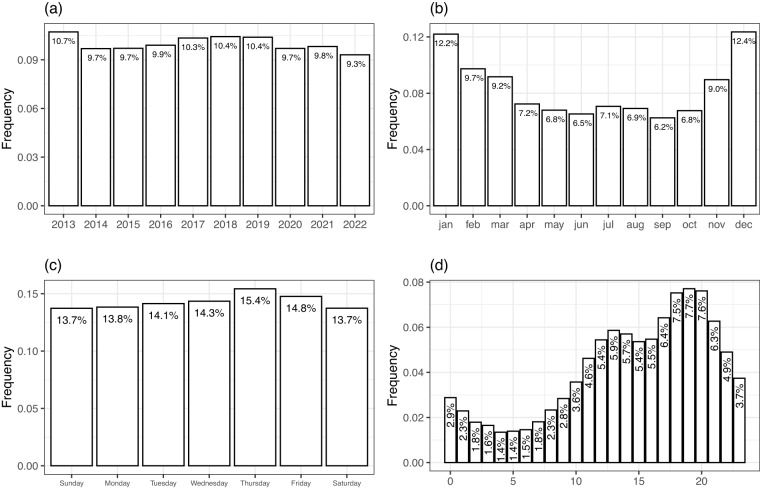
Distribution of urban fire occurrences (**a**) per year, (**b**) per month, (**c**) per weekday and (**d**) per hour of the day.

As mention previously one of the current main research trends focuses on the spatio–temporal analysis to study the patterns through which urban fires are distributed, structured and changed. Hence, the correctness of these two data components is of great importance. The data here made available pertains to the time frame between 2013 and 2022. During this decade, the number of urban fires per year was relatively uniform, ranging from 6764 in 2022 to 7784 in 2013, with an annual average of 7264 per year (Fig. 4a). Figure 4b shows a clear annual pattern, with the number of urban fires typically higher between November and March (≥9% of total events), corresponding to the autumn and winter seasons. Throughout the weekdays the distribution is nearly uniform (Fig. 4c), with a mild higher incidence by the end of the week (Thursday and Friday) and lower values during the weekend. Figure 4d shows that most of the occurrences happen during late afternoon and night-time hours, more precisely between 5 pm and 9 pm.

**Fig. 5:**
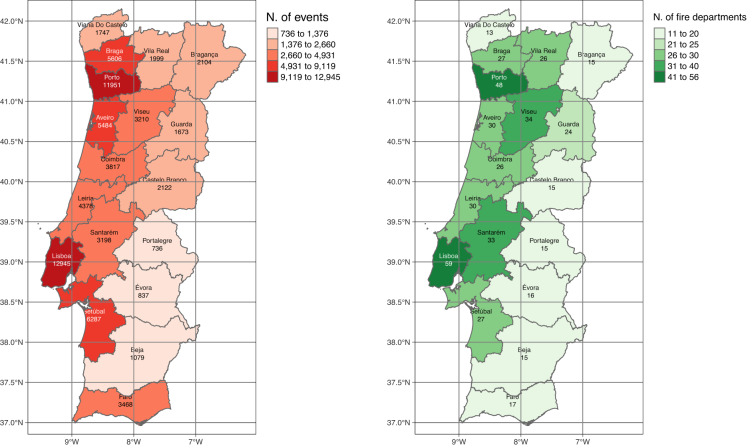
Total number of urban fire occurrences by district (left map) and total number of fire departments by district (right map).

Figure 5 shows the spatial distribution of urban fires and fire departments per district. Clearly there is an uneven distribution of fire incidents across Portugal, with a higher frequency along the coast. As expected^?^, Lisboa and Porto districts which are characterized by having among the highest population densities in Portugal, lead the incidence ranking of urban fires across districts. The pattern displayed by the number of fire departments closely mirrors this asymmetry.

**Fig. 6:**
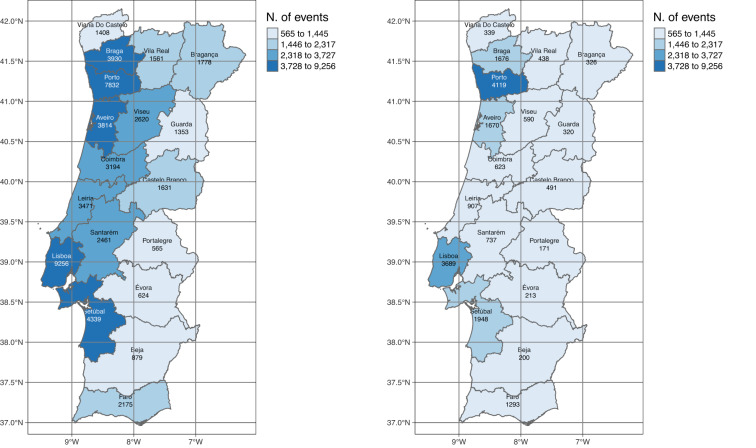
Distribution of the number residential (left map) and non-residential occurrences by district (right map).

The vast majority of urban fires occur in residential buildings, representing around 73% of all the events between 2013 and 2022. Among the fires occurring in non-residential urban infrastructures, the top three categories include *Industry, Workshops and Warehouses*, *Degraded or Unoccupied Buildings* and *Hotels and Restaurants*, each one representing a percentage above 5%. *Commercial areas and Transport stations* represent around 2% of the non-residential fire occurrences. Each one of the remaining type of structures represent less than 1% of the events. The events, occurring in residential infrastructures or not, are mainly observed in the major districts of Lisboa and Porto (Fig. 6). Lisboa and Porto districs aggregate around 18% and 16% of the residential events, respectively. The percentage of residential fires that were observed in the remaining districts ranged between approximately 1 (in Portalegre) to 9% (in Setúbal).

**Fig. 7:**
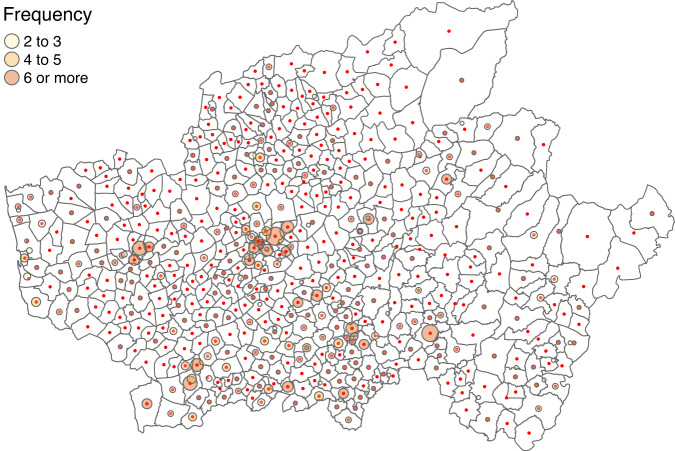
Braga district map showing the parishes boundaries. Red dots represent parishes centroids. Bubbles in the map represent and have size proportional to the number of recorded fires in the same location. Note that bubbles are centred around the centroids.

While analysing the frequency of fires spatial coordinates, we found that several entries presented exactly the same location (see, as an example, Fig. 7). In many cases, these duplicates occurred because spatial coordinates were not taken exactly but, instead, were endorsed by the parish centroid. Thus, a new categorical variable was added to the dataset–coordinates type (cty variable)–with categories *not exact* (representing 7% of the cases), if the spatial coordinates of the event matched the parish centroid or *exact* (93%), otherwise. As a consequence, two new validation rules were added (regarding variable cty, [${r}_{68}$] class attribute = factor and [${r}_{69}$] levels in code list, Table 3). Note that this procedure ensured the dataset to be suitable, as is, to a parish-area level analysis or a point process level analysis, if filtered by this new variable, retaining only, known to be, exact locations.

Around 10% of the events were categorized as having low importance. Approximately 40% and 50% were considered has medium or high importance urban fires. Regarding the fires length, half of the occurrences had a duration under approximately 1 hour and only 10% surpassed 2.3 hours. On average, the occurrences lasted around 1.4 hours.

The dataset also contains important information regarding human and technical resources. Around 24% of the events require less than 5 firefighters and most of them (44%) demanded between 5 and 10 ground human resources. In 1% of the events more than 35 firefighters were necessary to fight the occurrence. Most of the events took between 1 to 3 technical resources (69%). Aerial resources (human or technical) were used in approximately 0.2% of the events.

Additional information about deaths and injuries in urban fire occurrences is equally available in the dataset. In approximately 99% of the occurrences, there were no major injuries, while this value decreased to 93% when considering minor injuries. It should also be noted that 99.6% of the events had no fatal victims.

The above summary description purported to screen data correctness and facilitate data reuse by others. In addition, it may lift the veil on the potential that these data have towards the understanding of the phenomenon of urban fires in Portugal and/or serving as a tool for comparison with the reality in other countries. It should be noted that the provided summary description does not exhausts, nor intended to exhaust, the information retrievable from the dataset or the insights that may arise from cross-referencing the dataset variables with other sources of information (e.g., social-demographic, financial).

## Usage Notes

There is a wide array of possible usages based on the dataset now made available that may support a variety of urban firefighting decision-making mostly related with urban fire risk management and risk analysis aiming mitigation. The prime, and perhaps the most direct usage for this dataset, is, through the use of proper statistical methods, allow to map the probability of urban fire occurrence per location, with the data potentially supporting different geographical ranges, meaning different granularity, including parish, municipality, district, subregion and region levels. Furthermore, the combination of the probability of occurrence with the hazards associated with the events (e.g., injuries, deaths) may enable the creation of a full risk map (as a function of the probability of occurrence and local hazard). Within the scope of the probability of occurrence of fire events, the application of machine learning algorithms may further support the prediction of future fire events. Beyond the elaboration of risk maps, the dataset may be used for optimization of (*i*) geographical location of fire departments; (*ii*) resource allocation (human firefighters, vehicles, finance) across, e.g., the hundreds of fire departments, cities, districts, regions, and (*iii*) service area delimitation. Hence, at the policy level, the dataset can be used to rethink and optimize location and resources at local, regional or national levels. Finally, the dataset may be used to contribute to the fire departments performance assessment, using techniques as Data Envelopment Analysis (DEA) or machine learning models, allowing national authorities to evaluate the efficiencies of the fire departments, identify the most efficient and the areas for improvement on the inefficient units.

In addition to these main research usages, the dataset can also serve societal development goals including, e.g., data-driven journalism promoting population literacy based on open-access information principles and teaching/learning purposes where, for example, the data may be used in statistics advanced courses to exemplify spatio–temporal analysis, either considering a point process or area data.

## Data Availability

Chasing reproducibility, the code used in this study is openly accessible. The code is organized in two files: 1. SValidation.md, containing the code for reading the raw data[?], data transforming, data cleaning and structural validation actions, and; 2. CValidation.md, containing the code related to content validation procedures, which follow the actions taken on the former code file. These files are available on GitHub at https://github.com/rb1970/UrbanFiresData.
